# The Invisible Excess: Too Long Antibiotic Duration in the Pediatric Emergency Care

**DOI:** 10.3390/antibiotics15020128

**Published:** 2026-01-27

**Authors:** Miguel Ángel Molina-Gutiérrez, María Camacho-Gil, Virginia Santana-Rojo, Luis Escosa-García

**Affiliations:** 1 Pediatric Emergency Department, La Paz University Hospital, 28046 Madrid, Spain; miguelangel.molina@salud.madrid.org; 2 Department of Pediatric Internal Medicine, La Paz University Hospital, 28046 Madrid, Spain; maria.camacho@salud.madrid.org (M.C.-G.); virginia.santana@salud.madrid.org (V.S.-R.); 3 Pediatric Infectious Diseases Department, La Paz University Hospital, 28046 Madrid, Spain; 4 Área de Enfermedades Infecciosas, Centro de Investigación Biomédica en Red de Enfermedades Infecciosas (CIBERINFEC), Instituto de Salud Carlos III, 28029 Madrid, Spain

**Keywords:** antimicrobial stewardship, pediatric emergency medicine, duration of therapy, community-acquired pneumonia

## Abstract

**Background/Objectives**: Antibiotics are among the most commonly prescribed medicines in the Pediatric Emergency Department (PED). The overuse of antibiotics is directly linked to the emergence of resistance. Recent clinical trials have emerged in children in which short courses have proven to be as effective as longer courses. The aim of this study was to analyze the duration of antibiotic treatment prescribed in our PED for the most important and common infections in children and to compare with the best available evidence. **Methods**: A single-center retrospective study was conducted in the PED of a tertiary hospital. We evaluated outpatients from birth to 16 years who were discharged with antibiotic therapy during a 1-year period (2022) to classify duration of therapy as appropriate or inappropriate. **Results**: 1972 antibiotic prescriptions were analyzed. 28.3% (560/1972) of the prescriptions were classified as inappropriate according to duration of therapy; 551 (98.3%) were due to longer-than-recommended duration. The condition associated with the highest number of inappropriate prescriptions was Uncomplicated Community-acquired Pneumonia (CAP) (427/560; 76.2%). When focusing on each infectious syndrome, Uncomplicated CAP had also the highest percentage of inappropriate duration (92.6%) comparing with appropriate prescription. Regarding specific types of antibiotics, amoxicillin accounted for the highest number of inappropriate prescriptions (422/560; 75.4%). **Conclusions**: A longer-than-recommended prescription of antibiotics is frequent in the Pediatric Emergency Department. Uncomplicated CAP is the condition associated with the highest number of inappropriate duration of antibiotics in our setting.

## 1. Introduction

Over the past 80 years, antibiotics have emerged as a crucial component in the management of infectious diseases. However, the use and access to antimicrobial drugs has become more widespread because of the evolution of health care systems, leading to an increase in their inappropriate use [1,2]. Antibiotics are among the most commonly prescribed medicines for infants and children, although recently early exposure has been linked to adverse long-term health outcomes (allergies, asthma, obesity, neurodevelopmental disorders, etc.) [3]. In various settings, more than two-thirds of children receive antibiotics before the age of two. On average, more than half of all children receive at least one antibiotic per year. The highest rate of antibiotic prescribing is observed in the second year of life [4,5]. The high rate of antibiotic prescribing is largely because children are more susceptible to infections and illnesses that often require antibiotic treatment. This special vulnerability of children is one of the main arguments used to pressure pediatricians to prescribe antibiotics in many situations in which these drugs are not really necessary [6]. The pediatric emergency setting is one of the scenarios where such pressure is even greater, and it is not easy for professionals to align patients and relatives in the rational use of these drugs.

The overuse of antibiotics is directly linked to the emergence of resistance [7,8,9]. Antimicrobial resistance (AMR) is a major public health problem and the leading cause of morbidity and mortality from previously treatable infections [10]. Recognizing the severity of AMR, the World Health Organization (WHO) has ranked it among the ten global threats to health worldwide, calling for the prudent use of antibiotics [11]. When optimizing the use of antibiotics, a key strategy is to reduce the treatment duration. However, for certain infections, the opposite has occurred [12].

Classically, the choice of the average duration of antibiotic treatment has been based on clinical experience, estimated from those for adults or from non-evidence-based old practices. Over the past 25 years, >100 clinical trials have demonstrated that short-course antibiotic treatment is non-inferior to classical longer therapy in adults, with few exceptions [13]. Also, recent clinical trials have emerged in children in the last few years [14,15,16,17,18] in which short courses have proven to be effective. Fewer adverse effects and less risk of resistance selection are both related to shorter therapy; those strategies are also more economical and favor adherence to treatment, which is essential in the pediatric population.

The aim of this study was to analyze the duration of antibiotic treatment prescribed in our Pediatric Emergency Department (PED) for the most important and common infections in children and to compare with the best available evidence for each infectious syndrome according to international guidelines. This intervention is an exploratory work in our hospital in the context of an antimicrobial stewardship program focusing specifically on improving practices on duration of antimicrobial therapy.

## 2. Results

In 2022, there were 54,583 visits to our PED. Of these patients, 1972 antibiotic prescriptions were analyzed (3.6%). The median age of the patients was 3.2 years (interquartile range (IQR): 1.6–5.5). Regarding gender distribution, 51.7% (1020/1972) were male and 48.3% (952/1972) were female. Table 1 shows the personal medical conditions of the patients.

Of the antibiotic prescriptions analyzed, 28.3% were for acute otitis media (AOM) in children older than two years, 23.4% for uncomplicated community-acquired pneumonia (CAP), 20.3% for AOM in children younger than two years, and 18.3% for acute streptococcal pharyngitis (Table 2).

**Figure 1:**
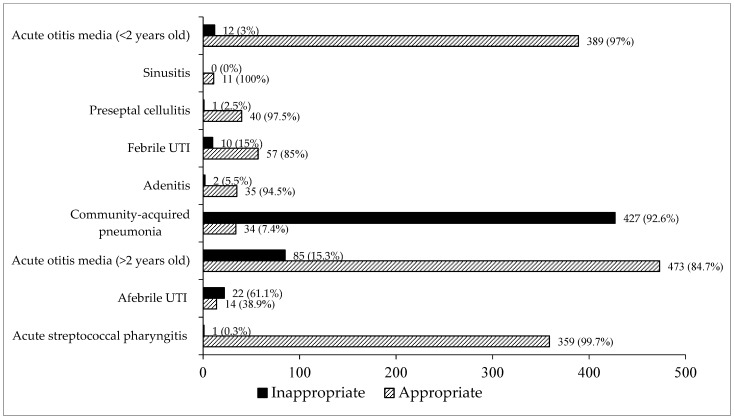
Total number of prescriptions according to infectious disease and evaluation of duration of therapy.

Overall, 28.3% (560/1972) of the prescriptions were classified as inappropriate according to duration of therapy. Of these, 551 (98.3%) were due to longer-than-recommended duration of therapy, while 9 (1.6%) corresponded to shorter-than-recommended duration. The condition associated with the highest number of inappropriate prescriptions was uncomplicated CAP (427/560; 76.2%), followed by AOM in children older than two years (85/560; 15.2%). When focusing on each infectious syndrome, uncomplicated CAP (92.6%) and afebrile urinary tract infection (UTI) (61.1%) were those with the highest percentages of inappropriate duration comparing with appropriate prescription (Figure 1). The actual distribution of prescribed antibiotic durations for each infectious syndrome, including median values, interquartile ranges (IQR), and overall ranges, is shown in Table 3.

Regarding specific types of antibiotics prescribed in the PED (Table 4), amoxicillin was the most frequent (64%), followed by amoxicillin–clavulanic acid (16.6%) and phenoxymethyl penicillin (10.5%). Amoxicillin accounted for the highest number of inappropriate prescriptions (422/560; 75.4%), followed by amoxicillin–clavulanic acid (96/560; 17.1%), cefixime (22/560; 3.9%), cefuroxime (16/560; 2.0%), levofloxacin (2/560; 0.4%), and phenoxymethyl penicillin (1/560; 0.2%). Among children diagnosed with AOM (*n* = 959), amoxicillin was the most frequently prescribed antibiotic, both in those under two years of age (291/401; 72.6%) and in older children (461/558; 82.6%). In both age groups, amoxicillin was also the antibiotic associated with the highest proportion of inappropriate prescriptions, with statistically significant differences observed (58.3%, *p* = 0.011 and 68.2%, *p* = 0.004, respectively). In absolute terms, amoxicillin accounted for the highest number of prescriptions with inappropriate duration, largely reflecting its predominant use in high-volume conditions such as acute otitis media. However, when proportions were calculated within each antibiotic group, the proportion of inappropriate duration in children older than two years with AOM was higher among those receiving amoxicillin–clavulanic acid compared with amoxicillin (29.3% vs. 12.6%), as shown in Table 4 and Table 5.

Similarly, among children diagnosed with uncomplicated CAP (*n* = 461), amoxicillin was again the most commonly prescribed antibiotic (367/461; 79.6%). Amoxicillin accounted for the highest absolute number of inappropriate prescriptions in this group. The proportion of inappropriate prescriptions was significantly higher among those who received amoxicillin (83.6%, *p* < 0.001) (Table 5), but this finding reflects its widespread use rather than a higher relative risk compared with other antibiotics.

In the group of children diagnosed with UTIs, regardless of the presence of fever, the highest proportion of inappropriate prescriptions was observed for cefixime (Table 5).

According to seasonality (Supplementary Information, Figure S1), fluctuations were not statistically significant (*p* = 0.375). Further analysis by season (spring, summer, autumn, and winter) also yielded no significant differences (*p* = 0.208), suggesting a stable distribution throughout the year.

## 3. Discussion

In this study, conducted in a tertiary PED setting, 1972 pediatric patient records from 2022 were analyzed to evaluate the duration of antibiotic therapy for the most common ambulatory infections diagnosed in the PED. Nearly one-third of the antibiotic prescriptions were classified as inappropriate in terms of duration, based on the best available evidence (Supplementary Information, Table S1). Among all the conditions assessed, uncomplicated CAP had, by far, the highest proportion of inappropriate prescriptions (92.6%). In a mini-review of studies prepared by the authors where duration of antimicrobial therapy was specifically evaluated in the PED (Table 6) we concluded that durations longer than minimally recommended was as frequent as 36.8% in contrast with 28.3% established in our PED [19,20,21,22,23,24]. Krueger C et al. also documented that CAP was the infection with the highest percentage of prescriptions given with durations longer than minimally recommended when comparing with UTI or AOM (54.5% vs. 44–47%) [21].

Recent data of clinical trials in children in the last few years [14,15,16,17,18] should encourage reflection in the environment of PED. Infectious diseases are among the leading causes of visits. Previous studies report that infectious diseases account for approximately 28% of all PED visits [25]. In Spain, other research estimates this figure to be even higher, reaching up to 45% of PED visits due to infectious diseases [20]. Among these, upper respiratory tract infections are the most common, representing 40.8% of infectious disease-related PED visits [25]. Given the significant prevalence of infectious diseases in pediatric emergencies, it is reasonable to infer that antibiotics are frequently prescribed in this context. Studies in pediatric populations indicate that antibiotics are prescribed at discharge in 25.5% of visits for infectious diseases, with amoxicillin being the most commonly used antibiotic, prescribed in 60.6% of these cases [20]. Moreover, global inappropriate antibiotic prescribing occurs in 22.9% of pediatric cases, compared to 36.9% in adults. Some studies attribute this difference to the relative simplicity of therapeutic decisions in children, due to fewer comorbidities, as well as a tendency to prescribe antibiotics more cautiously in the pediatric population [26].

In our series, pneumonia was the condition with the highest rate of patient discharge associated with inappropriate antibiotic prescribing. Recent meta-analyses have demonstrated that short-term treatment with amoxicillin is as effective as long-term treatment for uncomplicated pneumonia in children under 10 years of age [27]. In this context, optimizing treatment in the PED is particularly important, as there are currently no rapid diagnostic tools to identify the specific etiology of suspected bacterial lung infections. Therefore, optimizing the duration of empirical treatment for uncomplicated CAP (5 days) represents a significant opportunity for improvement and should be a priority for emergency physicians [28]. We also documented that a frequent indication for antibiotics in PED, AOM in children > 2 years, had a high proportion of inappropriate prescriptions (15.3%). This was lower than prescriptions given with durations longer than minimally recommended by Krueger C et al. [21] (46.7%), but we can not compare strictly as we considered 5–7 days as an appropriate duration in contrast with the Canadian study where AOM > 2 years duration was established as 5 days.

Several barriers may contribute to the observed lack of adherence to evidence-based recommendations regarding antibiotic duration in the pediatric emergency setting. First, parental expectations and pressure to prescribe antibiotics, particularly for respiratory infections in young children. Clinicians may respond to these expectations by favoring longer treatment courses as a perceived safety margin, even when shorter durations are supported by evidence. Second, diagnostic uncertainty is inherent to the emergency department environment, especially for syndromes such as community-acquired pneumonia, where etiology is often presumptive, clinical presentation may overlap with viral infections, and access to rapid diagnostic tools or follow-up radiography is limited. In this context of uncertainty, extending antibiotic duration may be viewed as a risk-mitigation strategy to avoid potential treatment failure. Third, challenges related to outpatient follow-up—including limited access to timely reassessment, variable health literacy among caregivers, and concerns about loss to follow-up after emergency discharge—may further reinforce the tendency toward longer-than-recommended courses. These barriers underscore the need for tailored antimicrobial stewardship interventions in the emergency setting, integrating clinician education on current evidence, structured caregiver communication strategies to manage expectations, decision-support tools to reduce diagnostic uncertainty, and system-level improvements to ensure safe follow-up after shorter treatment courses.

Beyond general awareness campaigns, our findings support the implementation of targeted, actionable antimicrobial stewardship interventions tailored to the pediatric emergency department setting. Given that uncomplicated community-acquired pneumonia accounted for over three-quarters of inappropriate prescriptions in our study, priority should be given to syndrome-specific interventions with the greatest potential impact. First, incorporating evidence-based default antibiotic durations into electronic prescribing systems—particularly for high-volume syndromes such as uncomplicated CAP (5 days) and acute otitis media in children older than two years (5–7 days)—could serve as a simple yet effective nudge toward guideline-concordant prescribing. Second, standardized discharge order sets with pre-populated, evidence-based treatment durations and syndrome-specific clinical pathways can reduce variability and cognitive burden during high-pressure clinical encounters. Third, integrating brief, non-intrusive decision-support prompts or alerts at the point of prescription—triggered when durations exceed evidence-based recommendations—may provide real-time guidance without disrupting workflow. Fourth, equipping caregivers with standardized, literacy-appropriate written materials that explain the rationale for shorter antibiotic courses, address common misconceptions, and provide clear instructions for follow-up can help align parental expectations and improve treatment acceptance. Finally, establishing periodic audit and feedback mechanisms focused specifically on treatment duration—rather than antibiotic selection alone—with individualized prescriber-level data and peer comparisons, could reinforce adherence to current evidence and sustain practice change over time. These multilevel interventions, addressing system, prescriber, and caregiver factors simultaneously, are more likely to achieve meaningful and durable improvements in antibiotic prescribing practices.

Afebrile UTI in our study had an important percentage of inappropriate prescriptions according to duration (61%) and was the only infectious syndrome, together with uncomplicated CAP, where inappropriate durations were more frequent than appropriate ones. Focusing on febrile UTI, 15% of our patients had a longer-than-recommended duration of therapy; in this scenario, a 7–10-day course of antibiotics is usually recommended [29,30,31,32]. Several studies have shown that more than 90–95% of children with febrile UTI are afebrile and clinically better 48–72 h after initiation of treatment [33,34,35]. Our center is a national reference hospital in pediatric nephrology and has a special unit for complex chronic patients, which is constantly expanding. Therefore, a significant percentage of UTI observed in our PED patients occurs in children with nephro-urological pathology, complex chronic pathology, and those who have undergone kidney transplantation. Although our work excluded children with this type of antecedents, it seems remarkable to point out the problem of adjusting the duration of empirical antibiotherapy in this profile of patients, since in many cases these infections are produced by multiresistant microorganisms that could condition the therapeutic scheme.

This study has several limitations that warrant consideration. First, due to its retrospective design, clinical outcomes such as treatment failure, recurrence, or adverse events were not systematically captured or assessed. Consequently, we were unable to directly evaluate whether longer-than-recommended antibiotic courses resulted in different patient-level outcomes compared to guideline-concordant durations. However, it is important to note that the recommended durations used as benchmarks in this study were derived from high-quality randomized controlled trials and evidence-based international guidelines that have consistently demonstrated non-inferiority—and in some cases, equivalence—of shorter courses for the infectious syndromes evaluated. Thus, the classification of prescriptions as inappropriate was grounded in robust clinical evidence rather than arbitrary standards. Second, data on prescriber characteristics—including level of training, years of clinical experience, specialty, and shift timing—were not available in the medical records reviewed. This precluded analysis of provider-level factors that may influence prescribing behavior and represent important targets for stewardship interventions. Third, as a single-center study conducted in a tertiary PED, external validity may be limited, and findings may not fully reflect prescribing practices in community hospitals, primary care settings, or emergency departments with different patient populations or resource availability. Nonetheless, the prescribing patterns observed—particularly the high rate of prolonged treatment for uncomplicated CAP—are consistent with those reported in other pediatric emergency settings, suggesting that our findings may have broader relevance.

## 4. Materials and Methods

A single-center retrospective study was conducted in the PED of a tertiary hospital, with a mean of visits of 50,000–55,000 per year. The sample included pediatric outpatients aged from birth to 16 years who were discharged with antibiotic therapy during a 1-year period (January 2022–December 2022).

Data were collected for this study by medical chart review. Patients were identified consecutively using the HCIS (Health Care Information System) diagnostic search system. We used the HCIS diagnostic search system to select all pediatric patients diagnosed in the PED with: afebrile UTI, febrile UTI, preseptal cellulitis, acute lymphadenitis, AOM, acute sinusitis, acute streptococcal pharyngitis, and uncomplicated CAP. Uncomplicated CAP was defined as a clinical diagnosis of pneumonia made in the PED in children discharged with oral antibiotic therapy, without criteria for hospital admission. Cases were considered uncomplicated when there were no signs of severe disease (such as hypoxemia, respiratory distress requiring oxygen therapy, hemodynamic instability, or altered level of consciousness), no radiological or clinical complications (including pleural effusion or empyema), and no significant underlying conditions predisposing to severe infection.

Diagnoses were based on the attending physician’s clinical diagnosis recorded in the electronic medical record at the time of discharge. Patients were excluded from the study if these conditions were present: immunocompromised patients, UTI in children with a history of severe genitourinary tract disease, acute lymphadenitis with abscess, AOM in patients with tympanostomy tubes or cochlear implants, and acute streptococcal pharyngitis associated with local complications (retropharyngeal/peritonsillar abscess).

Medical chart review was performed by two investigators using a standardized data extraction form. Discrepancies were resolved by consensus. Data collected included age at presentation, sex, personal medical conditions, type of infection, type of antibiotic and duration (days). To classify as an appropriate or inappropriate duration of therapy, we used previous standard practice recommendations adapted from: (1) The Australian and New Zealand Paediatric Infectious Diseases Group; (2) Recent data from 2021–2024 UTI and CAP clinical trials in children (including unpublished data at that moment) [36,37] (Supplementary Information, Table S1). Duration of therapy was classified by comparing the prescribed duration with predefined ranges for each condition; treatments shorter or longer than the recommended range were considered inappropriate. Appropriate duration was: afebrile UTI 3–4 days, febrile UTI 7–10 days, preseptal cellulitis 7–10 days, acute lymphadenitis 5–10 days, AOM (<2 years old) 7–10 days, AOM (>2 years old) 5–7 days, acute sinusitis 7–10 days, acute streptococcal pharyngitis 5–10 days, and uncomplicated CAP 5 days. We also explored the potential seasonality of inappropriate duration by comparing monthly proportions and by season.

Results are presented as absolute frequencies and/or percentages; quantitative data are expressed as median and IQR. Categorical variables were compared using the chi-squared test or Fisher’s exact test. *p*-values less than 0.05 were considered significant. All analyses were performed with the Statistical Package for the Social Sciences (IBM SPSS Statistic Version 21, IBM Inc., Chicago, IL, USA). The study was approved by the local research ethics committee (PI-6193).

## 5. Conclusions

A longer-than-recommended prescription of antibiotics is frequent in the Pediatric Emergency Department. Uncomplicated Community-acquired Pneumonia is the condition associated with the highest number of inappropriate duration of antibiotics in our setting. The results of our study show that, although there is increasing evidence in favor of shortening the duration of antibiotic treatment, we are still far from achieving a real change in routine clinical practice in pediatric emergency care. Therefore, we believe that it is essential to conduct awareness campaigns on the rational use of antibiotics in an emergency setting so that they are as close as possible to the most recent recommendations on the duration of treatment.

## Figures and Tables

**Table 1:** Personal medical conditions of patients in this study.

	N (%)
Gastrointestinal disorders	38 (1.9)
Hemato-Oncology disorders	24 (1.2)
Neurological disorders	57 (2.9)
Chromosomal disorders	33 (1.7)
Cardiological disorders	26 (1.3)
Pulmonary disorders	150 (7.6)
ENT	61 (3.1)
Nephrourology conditions	45 (2.3)
Allergy to macrolides	3 (0.2)
Allergy to beta-lactams	11 (0.6)
History of UTI	62 (3.1)
History of AOM	62 (3.1)
History of pneumonia	30 (1.5)

ENT: Ear, Nose and Throat. UTI: Urinary Tract Infection. AOM: Acute Otitis Media.

**Table 2:** Infectious diseases of patients in this study.

	N (%)
Acute otitis media (<2 years old)	401 (20.3)
Acute otitis media (>2 years old)	558 (28.3)
Acute streptococcal pharyngitis	360 (18.3)
Adenitis	37 (1.9)
Community-acquired pneumonia	461 (23.4)
Febrile UTI	67 (3.4)
Afebrile UTI	36 (1.8)
Preseptal cellulitis	41 (2.1)
Acute sinusitis	11 (0.6)
Total	1972

UTI: Urinary Tract Infection.

**Table 3:** Distribution of prescribed antibiotic duration by infectious syndrome (days).

Infectious Syndrome	Median (IQR)	Range (Min–Max)
AOM (<2 years)	7 (7–7)	(3–21)
AOM (>2 years)	7 (7–7)	(3–21)
Acute streptococcal pharyngitis	10 (10–10)	(0–10)
Adenitis	7 (7–10)	(7–14)
CAP	7 (7–7)	(0–14)
Preseptal cellulitis	7 (7–10)	(5–10)
Febrile UTI	10 (10–10)	(1–14)
Afebrile UTI	5 (1–7)	(1–10)
Acute sinusitis	10 (7–10)	(7–10)

AOM: Acute otitis media. CAP: Community-acquired pneumonia. SD: Standard deviation. IQR: Interquartile range.

**Table 4:** Antibiotic prescriptions by infectious disease and antibiotic class, *n* (%).

	Penicillins			Cephalosporins				Macrolides		Others			
	AMOX (*n* = 1264)	AMOX/CLAV (*n* = 327)	PEN V (*n* = 207)	CEFDX (*n* = 10)	CFM (*n* = 78)	CRO (*n* = 10)	CFX (*n* = 24)	AZM (*n* = 31)	CLR (*n* = 2)	FOS (*n* = 15)	CLIN (*n* = 1)	DOX (*n* = 1)	LVX (*n* = 2)
**AOM** **(<2 years old)**	291 (72.6)	92 (22.9)	1 (0.2)	-	5 (1.2)	5 (1.2)	4 (1)	2 (0.5)	1 (0.2)	-	-	-	-
**AOM** **(>2 years old)**	461 (82.6)	82 (14.7)	-	-	3 (0.5)	5 (0.9)	3 (0.5)	3 (0.5)	1 (0.2)	-	-	-	-
**ASP**	136 (37.8)	13 (3.6)	206 (57.2)	-	1 (0.3)	-	2 (0.6)	2 (0.6)	-	-	-	-	-
**Adenitis**	2 (5.4)	23 (62.2)	-	10 (27)	-	-	-	-	-	-	1 (2.7)	1 (2.7)	-
**CAP**	367 (79.6)	61 (13.2)	-	-	1 (0.2)	-	6 (1.3)	24 (5.2)	-	-	-	-	2 (0.4)
**Febrile UTI**	1 (1.5)	3 (4.5)	-	-	60 (89.9)	-	2 (3)	-	-	1 (1.5)	-	-	-
**Afebrile UTI**	-	7 (19.4)	-	-	8 (22.2)	-	7 (19.4)	-	-	14 (38.9)	-	-	-
**Preseptal** **cellulitis**	-	41 (100)	-	-	-	-	-	-	-	-	-	-	-
**Sinusitis**	6 (54.5)	5 (45.5)	-	-	-	-	-	-	-	-	-	-	-

AOM: Acute otitis media. ASP: Acute streptococcal pharyngitis. CAP: Community-acquired pneumonia. AMOX: Amoxicillin. AMOX/CLAV: Amoxicillin–clavulanic acid. AZM: Azithromycin. CEFDX: Cefadroxil. CFM: Cefixime. CLR: Clarithromycin. CRO: Ceftriaxone. CFX: Cefuroxime. PEN V: Phenoxymethyl penicillin (penicillin V). FOS: Fosfomycin. CLIN: Clindamycin. DOX: Doxycycline. LVX: Levofloxacin. UTI: Urinary Tract Infection.

**Table 5:** Comparison of cases of inappropriate duration based on the type of antibiotic and infectious disease.

	Total (*n* = 560) (%) *	Penicillins			Cephalosporins				Macrolides		Others				*p*-Value
		AMOX	AMOX/CLAV	PEN V	CEFDX	CFM	CRO	CFX	AZM	CLR	FOS	CLIN	DOX	LVX	
**AOM** **(<2 years old)**	12 (2.1)	7 (58.3)	2 (16.7)	0(0)	-	1 (8.3)	0(0)	2 (16.7)	0(0)	0(0)	-	-	-	-	**0.011**
**AOM** **(>2 years old)**	85 (15.2)	58 (68.2)	24 (28.2)	-	-	2 (2.4)	0(0)	1 (1.2)	0(0)	0(0)	-	-	-	-	**0.004**
**ASP**	1 (0.2)	0(0)	0(0)	1 (100)	-	0(0)	-	0(0)	0(0)	-	-	-	-	-	1
**Adenitis**	2 (0.4)	0(0)	1 (50)	-	0(0)	-	-	-	-	-	-	0(0)	1 (50)	-	0.138
**CAP**	427 (76.2)	357 (83.6)	61 (14.3)	-	-	1 (0.2)	-	6 (1.4)	0(0)	-	-	-	-	2 (0.5)	**<0.001**
**Febrile UTI**	10 (1.8)	0(0)	0(0)	-	-	10 (100)	-	0(0)	-	-	0(0)	-	-	-	1
**Afebrile UTI**	22 (3.9)	-	7 (31.8)	-	-	8 (36.4)	-	7 (31.8)	-	-	0(0)	-	-	-	**<0.001**
**Preseptal cellulitis**	1 (0.2)	-	1 (100)	-	-	-	-	-	-	-	-	-	-	-	-

AOM: Acute otitis media. ASP: Acute streptococcal pharyngitis. CAP: Community-acquired pneumonia. AMOX: Amoxicillin. AMOX/CLAV: Amoxicillin clavulanic acid. AZM: Azithromycin. CEFDX: Cefadroxil. CFM: Cefixime. CLR: Clarithromycin. CRO: Ceftriaxone. CFX: Cefuroxime. PEN V: Phenoxymethyl penicillin (penicillin V). FOS: Fosfomycin. CLIN: Clindamycin. DOX: Doxycycline. LVX: Levofloxacin. UTI: Urinary tract infection. * Percentage refers to the total number of inappropriate prescriptions.

**Table 6:** Mini-review of studies where duration of antimicrobial therapy was specifically evaluated in the Pediatric Emergency Department.

	Study Period (Months)	Antimicrobial Prescriptions (*n*)	Standard Practice (Guidelines)	Inappropriate by any Aspect	Durations Longer than Minimally Recommended	Comments
**Observational studies without intervention**						
**Hagedoorn NN** et al. 2020 [19]	16 m. (2017–2018)	7636	International	22.3% (973/4373)	20% (1525/7636)	Only oral prescriptions included. Inappropriate by any aspect referred only in uncomplicated respiratory infections and UTI.
**García Moreno FJ** et al. 2022 [20]	12 m. (2018–2019)	142	Local (Spain)	50.7% (72/142)	10.5% (10/95)	Duration evaluated only when indication and antibiotic election were appropriate.
**Krueger C** et al. 2024 [21]	48 m. (2018–2021)	10,609	National (Canada)	No data	48.3% (5131/10,609)	Only AOM >2 years, CAP and UTI were evaluated. Durations longer than minimally recommended: >7 days for UTI and CAP; >5 days for AOM.
**Petel DS** et al. 2025 [22]	12 m. (2022–2023)	1908	National (Canada)	56% (1072/1908)	44.9% (857/1908)	Only AOM and CAP were evaluated.
**Pre–post observational studies**						
**Hamner M** et al. 2022 [23]	12 m. (2019–2020)	2039	National (USA)	No data	40% (815/2039)	Only Pre-intervention data included. Only Skin and Soft Tissue Infections included.
**Silvestro E** et al. 2024 [24]	3 m. (2020)	1128	International	86.7% (879/1014)	18.8% (133/707)	Only Pre-intervention data included. Duration evaluated only when indication and antibiotic election were appropriate.
**Total**					**36.8%** (8471/22,994)	

UTI: Urinary Tract Infection. AOM: Acute Otitis Media. CAP: Community-Acquired Pneumonia.

## Data Availability

Due to privacy and ethical restrictions, the data used in this study are not publicly available. However, they are available upon reasonable request to the corresponding author, subject to privacy and data protection policies.

## Supplementary Materials

The following supporting information can be downloaded at: https://www.mdpi.com/article/10.3390/antibiotics15020128/s1, Table S1. Antibiotic duration standard practices for outpatients in the Pediatric Emergency Department (PED). Figure S1. Distribution by months of inappropriate vs. appropriate antibiotic duration in the Pediatric Emergency Department (PED). References [14,15,16,17,18,38] are cited in the Supplementary Materials.

## Abbreviations

The following abbreviations are used in this manuscript:

AMR	Antimicrobial resistance
AMOX	Amoxicillin
AMOX/CLAV	Amoxicillin clavulanic acid
AOM	Acute otitis media
ASP	Acute streptococcal pharyngitis
AZM	Azithromycin
CAP	Community-acquired pneumonia
CEFDX	Cefadroxil
CFM	Cefixime
CFX	Cefuroxime
CLIN	Clindamycin
CLR	Clarithromycin
CRO	Ceftriaxone
DOX	Doxycycline
ENT	Ear, Nose and Throat
FOS	Fosfomycin
HCIS	Health Care Information System
IQR	Interquartile range
LVX	Levofloxacin
PED	Pediatric Emergency Department
PEN V	Phenoxymethyl penicillin (penicillin V)
SD	Standard deviation
UTI	Urinary tract infection
WHO	World Health Organization
