# Supplementary material for: The Invisible Excess: Too Long Antibiotic Duration in the Pediatric Emergency Care
Source: Antibiotics (Basel). 2026 Jan 27;15(2):128. doi: 10.3390/antibiotics15020128 (PMC12937230; doi:10.3390/antibiotics15020128)

The Invisible Excess: Too Long Antibiotic Duration in the Pediatric Emergency Care.

Supplementary Information.

Figures/Tables

Tables:

Table S1. Antibiotic duration standard practices for outpatients in the Pediatric Emergency Department (PED).

Infectious Diseases in PED for outpatients	Antibiotic Duration Standard Practice (days)	Level of Evidence in children (ANZPID-ASAP in 2016)	New references of interest (since 2016)
**GENITOURINARY**			
**Afebrile UTI**	3/4	A-I	
**Febrile UTI**	7/10	**A-II/A-I**<br>**New relevant data**	- Zaoutis T, et al. JAMA Pediatrics. 2023.<br>- Montini G, et al. Pediatrics. 2024.
**SKIN AND SOFT TISSUE**			
**Preseptal cellulitis**	7/10	C-IV	
**Acute lymphadenitis**	5/10	D	
**ENT**			
**Acute streptococcal pharyngitis**	5/10	**B-I/A-I**<br>**New relevant data**	- Stahlgren GK, et al. BMJ. 2019.
**Acute otitis media (<2 years old)**	7/10 (when indicated)	-	
**Acute otitis media (>2 years old)**	5/7 (when indicated)	-	
**Acute bacterial sinusitis**	7/10 (when indicated)	D	
**PNEUMONIA**			
**Uncomplicated community-acquired pneumonia**	5	**A-I**<br>**New relevant data**	- Bielicki JA, et al. JAMA. 2021.<br>- Pernica JM, et al. JAMA Pediatr. 2021.<br>- Williams DJ, et al. JAMA Pediatr. 2022.

Figures:

Figure S1. Distribution by months of inappropriate vs. appropriate antibiotic duration in the Pediatric Emergency Department (PED).

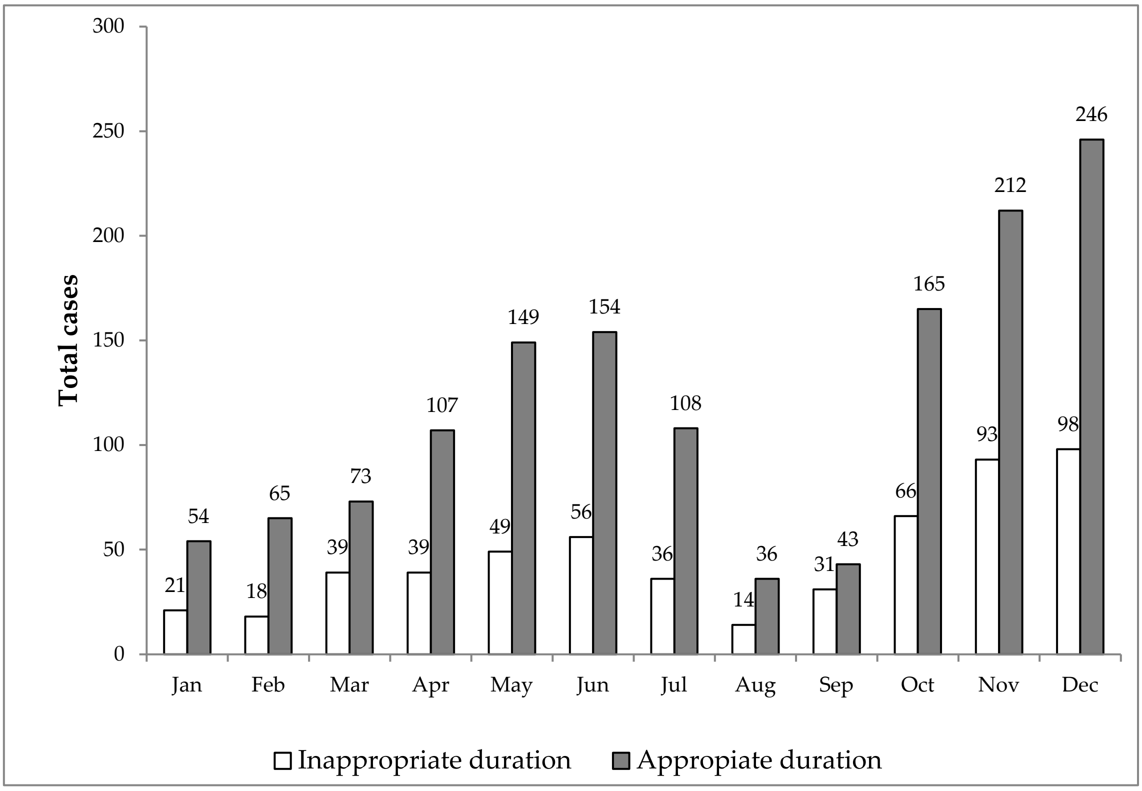

Supplement: Supplementary file 1 [file antibiotics-15-00128-s001.zip › antibiotics-4033628-supplementary.pdf]
